# Modeling the Influence of Macronutrients on the Heat Resistance of *Salmonella* in Milk Powder

**DOI:** 10.3390/foods14213757

**Published:** 2025-11-01

**Authors:** Xinyao Wei, Yi Lian, Shuxiang Liu

**Affiliations:** 1 College of Biological Science and Engineering, Fuzhou University, Fuzhou 350108, China; 2 Institute of Food Processing and Safety, School of Food Science, Sichuan Agricultural University, Ya’an 625014, China

**Keywords:** infant milk formula, dynamic thermal inactivation kinetics, dairy powder, microbial safety

## Abstract

Multiple *Salmonella* outbreaks linked to milk powders call for the need for effective pasteurization processes. Understanding the thermal inactivation kinetics of *Salmonella* in milk powder is crucial; however, the influence of macronutrient content (protein, fat, and carbohydrate) on these kinetics remains unclear. This study investigated the effects of common reconstitution temperature (45, 70, and 99 °C) on *Salmonella* survival in infant milk powder to raise public awareness about contamination risks. Seven milk powders with varying macronutrient compositions were used as model systems. After equilibrating to a uniform water activity (a_w_ = 0.2), the thermal resistance of *Salmonella* was determined at 75, 80, and 85 °C. The goodness-of-fit of two primary models (log-linear and Weibull) was compared, and secondary response surface models were developed to predict the combined effects of temperature and macronutrient composition on *Salmonella* inactivation. Results showed that *Salmonella* could proliferate or be resuscitated when contaminated milk powder was reconstituted at conventional preparation temperatures (45 °C and 70 °C). Across the seven formulations, *Salmonella* thermal resistance (*D*-value) increased with protein content (10.44–90.18%) and decreased with carbohydrate content (0.35–63.24%). A significant protein–temperature interaction was observed, whereby the effect of protein content on the *Salmonella D*-value decreased as temperature increased from 75 to 85 °C. Fat content did not significantly affect thermal inactivation (*p* > 0.05). The log-linear model provided a better fit than the Weibull model in this study. Overall, this research quantifies how macronutrient composition impacts *Salmonella* thermal resistance and offers predictive models to improve pasteurization strategies for milk powder.

## 1. Introduction

In recent years, *Salmonella* contamination has posed a serious threat to the safety of low-moisture foods (LMFs), which are foods with water activity below 0.85 [1]. According to the Rapid Alert System for Food and Feed (RASFF), approximately 28.9% of reported foodborne *Salmonella* contamination cases between 2002 and 2025 were linked to LMFs [2]. Contaminated LMFs accounted for 21% of *Salmonella* outbreaks recorded by the U.S. Centers for Disease Control and Prevention (CDC) from 2007 to 2018 [3]. Milk powder, a typical LMF, is commonly used as an ingredient in many ready-to-eat foods (requiring no further cooking before consumption), such as confectionery [4], mixed beverages [5], seasonings [6], and nutrition bars [7]. However, milk powder is highly susceptible to *Salmonella* contamination, particularly due to *Salmonella*-harboring powder residues trapped in spray dryer wall cracks. Therefore, spray drying is usually not considered a microbial killing step. Besides, no processing step follows spray drying to pasteurize milk powders [8,9]. Poor hygiene, inadequate sanitation, and lack of process control also create opportunities for cross-contamination [10]. These pose a great threat to the microbiological safety of milk powder. In 2011, a *Salmonella* contamination incident in infant formula occurred in Spain, resulting in 285 infections. A similar outbreak affected at least 30 infants across 11 cities in France in 2018 [11]. Between 2020 and 2025, the RASFF reported six milk powder -related issues, with two cases being denied entry due to the detection of *Salmonella* [2].

Despite common assumptions, milk powder is not inherently safe from microbial hazards [12,13]. While its low water activity (a_w_) prevents bacterial proliferation, it simultaneously enhances the survival and thermal resistance of pathogens like *Salmonella*. This bacterium can persist for years in dry environments and becomes increasingly difficult to eliminate through heat as moisture levels decrease [14,15,16]. Recent *Salmonella* outbreaks linked to milk powder have exposed critical weaknesses in both industrial thermal processing and home preparation methods. Conventional hot-water reconstitution provides insufficient protection against these heat-adapted pathogens. Therefore, urgent research is needed to evaluate the impact of reconstitution conditions on the survival rate of *Salmonella* in milk powder and to develop reliable pasteurization techniques specifically designed for low-moisture dairy products.

The effectiveness of pasteurization in milk powder is influenced by multiple variables. Beyond a_w_, the thermal resistance of microorganisms in low-moisture foods is shaped by a combination of intrinsic and extrinsic factors. These include bacterial strain or serotype [17,18,19], inoculation methods [20,21], product geometry [22,23], product composition [9,24,25], and processing temperature [26]. Food composition—specifically the type and proportion of constituents—significantly affects thermal resistance parameters (D- and z-values). Zhang et al. [27] reported that the *D*-values of *Salmonella* Enteritidis on egg powder increased with increasing fat (0–56.7%, w.b.) and decreasing protein contents (83.59–31.81%, w.b.). Rachon et al. [12] found that at higher temperatures, the 5 log reduction time for *Salmonella* was slightly greater in confectionery than in chicken flour, indicating that sugars provide stronger heat protection. Certain compounds like lactose, mannitol, sucrose, and rhamnose are known to enhance *Salmonella* heat tolerance [9,28], whereas glucose may reduce it [28]. Previous studies have predominantly focused on examining the effects of one or two specific nutrients on *Salmonella* heat resistance in simplified low-moisture food matrices. However, the wide variety of milk powder products, such as whole milk powder, nonfat dry milk, whey protein powder, lactose-free infant milk, etc., which have different nutritional compositions may result in differences in bacterial heat resistance. This variability complicates the task for manufacturers who need to achieve consistent pathogen control while preserving nutritional quality across different product types. How macronutrients (proteins, fats, and total carbohydrates) in milk powder independently or synergistically influence *Salmonella* thermal resistance in combination with temperature remains an unresolved yet critical question for process design.

This research was designed to: (1) quantify the survival of *Salmonella* during the reconstitution of milk powder under various water temperatures; (2) define the role of macronutrients in shaping *Salmonella* inactivation kinetics; and (3) assess the synergistic effects of macronutrients and temperature on *Salmonella* thermal inactivation.

## 2. Materials and Methods

### 2.1. Milk Powder Products

Seven types of commercially sourced milk powder, including whole milk powder (WMP), nonfat dry milk (NFDM), whey protein powder (WPP), casein protein powder (CPP), infant milk powder (IMP), lactose-free infant milk (LFIM), and amino acid-based formula (AAF), were selected for this study. All samples, representing different production lots, were procured from Nestlé China Ltd. (Beijing, China). Upon arrival, each sample underwent proximate analysis and background microorganism testing according to the method of Wei et al. [14]. The composition of the seven powders is shown in Table 1. Additionally, the water activity of all samples was measured at 25 °C using a water activity meter ((HD-7, Wuxi Huake Instrument Co., Ltd., Wuxi, China).

### 2.2. Proximate Composition Analysis

Compositional analysis of the seven milk powder samples was performed using official methods [29]: ash (method 930.30), fat (method 932.06), protein (method 930.29), and moisture contents (method 927.05). The carbohydrate content was obtained by subtracting the sum of all other constituent weights from the total.

### 2.3. Bacterial Cultures and Inoculation Procedure

Procedures to prepare the *Salmonella* inoculum were similar to those of Wei et al. [14]. *Salmonella enterica* subsp. *enterica* Enteritidis PT30 was used in this study because of its relatively high tolerance to heat in LMFs [15,30]. The frozen bacterial stocks were maintained at −80 °C in trypticase soy broth (TSB; Hope Bio-technology Co., Ltd., Qingdao, China) with 0.6% (*w*/*v*) yeast extract (YE; Oxoid Limited, Basingstoke, UK) supplemented with 20% glycerol until use. After thawing a frozen *Salmonella* culture at 22 °C for 10 min, 1 mL was inoculated into 10 mL of TSBYE and incubated at 37 °C for 24 ± 2 h. A loopful of the overnight culture was streaked onto tryptic soy agar (TSA; Hope Bio-technology Co., Ltd., Qingdao, China) supplemented with 0.6% (*w*/*v*) yeast extract (YE). After incubation, a single isolated colony was transferred into 10 mL of TSBYE and incubated at 37 °C for 24 h for secondary enrichment. To prepare the inoculum, a 0.1 mL aliquot of the broth culture was subcultured onto TSAYE and incubated at 37 °C for 24 h. The resultant bacterial lawn was then suspended in 3 mL of 0.1% buffered peptone water (BPW; Hope Bio-technology Co., Ltd., Qingdao, China) with an L-shaped spreader. This procedure yielded a *Salmonella* inoculum with a final concentration of approximately 10^10^ CFU/mL. All sample inoculations were performed in a biosafety cabinet. Each of WMP, NFDM, WPP, CPP, IMP, LFIM, or AAF was weighed (100.0 ± 0.1 g) and placed uniformly on a sanitized aluminum tray. The seven milk powder samples were spray-inoculated with 10 mL of the prepared *Salmonella* inoculum, separately. Then, the milk powder was quickly stirred to promote uniform mixing with the suspension and prevent clumping of the milk powder.

### 2.4. Sample Equilibration and Storage Stability

To achieve a target a_w_ of 0.20 at 25 °C, the inoculated samples were placed in a custom-built controlled-humidity chamber where the relative humidity was maintained at 20%. Water activity is one of the key environmental factors affecting microbial growth. This operation provides an experimental model for studying the survival of *Salmonella* under low water activity conditions. At days 0, 1, 2, 3, 4, 5, 8, 10, 16, and 20, a 3 ± 0.1 g subsample was collected from each batch of inoculated powder and placed into a sterile homogenizing bag for enumeration. The enumeration method was based on the approach described by Wei et al. [31] with minor modifications. A 10-fold dilution was performed by adding 27 mL of 0.1% BPW to each sample, followed by 5 min of homogenization. Homogenized sample were serially diluted in 9 mL of 0.1% BPW.A 100 μL aliquot of the dilution was aspirated and inoculated in duplicate onto m-TSA (TSAYE supplemented with 0.05% ammonium iron citrate, and 0.03% sodium thiosulfate) and incubated for 24 h at 37 ± 2 °C. After incubation, colonies with a black center were enumerated as *Salmonella*.

### 2.5. Reconstitution Experiment of IMP

This study explores the impact of reconstitution with water at different temperatures (45 °C, 70 °C, and boiling water) on the survival of *Salmonella* inoculated on IMP. After 3 days of equilibration in the chamber, inoculated IMP was mixed with water at a fixed ratio of 14%, reflecting standard infant formula preparation. Temperature-controlled water was added directly to the powder in the beaker and immediately mixed using a magnetic stirrer for 30 s to ensure homogeneity. The reconstituted milk was placed at room temperature and microbiologically analyzed after 0, 2, 4, and 6 h to evaluate *Salmonella* contamination levels in IMP at different consumption time points.

### 2.6. Isothermal Treatment

Isothermal treatments were performed on inoculated milk powder equilibrated to a a_w_ of 0.20 ± 0.02 for 3 days using a custom thermal lethality testing device developed by Chung et al. [32]. The selected treatment temperatures of 75, 80, and 85 °C were determined through preliminary experiments to optimize between sample quality preservation and processing efficiency, as higher temperatures cause discoloration while lower temperatures require impractical processing durations. Each thermal death curve was established through six sampling time points designed to achieve a minimum 3-log reduction of *Salmonella* populations. Immediately following each thermal treatment, samples underwent rapid cooling in an ice-water bath for one minute to terminate thermal inactivation, with subsequent microbial enumeration conducted according to the previously detailed methodology for sample equilibration and stability assessment.

### 2.7. Primary Model Fitting

This study employed two primary inactivation models to describe the survival kinetics in milk powders: the log-linear model (1) and the Weibull-type model (2), following the methodology of Peleg and Cole [33]:

(1) $$\log_{10}\left(\frac{N}{N_{0}}\right)=-\frac{t}{D_{T}}$$

(2) $${log}_{10}(\frac{N}{N_{0}})=-(\frac{t}{\beta }{)}^{\alpha }$$

The log-linear model parameters were defined as follows: N and $N_{0}$ (CFU/g) represent the concentration of *Salmonella* at time t and time zero, respectively; t (min) is the treatment duration; and $D_{T}$ (min) denotes the decimal reduction time at temperature *T* (°C); β is the scale parameter representing the overall steepness of the survival curve, where β approaching 1 indicates the sterilization process is approximately log-linear, while β deviating from 1 suggests a complex decontamination process. The shape parameter α determines the curve’s concavity: α > 1 produces downward concavity, α < 1 creates upward concavity, and α = 1 represents approaching a straight line, reverting to the first-order kinetic model.

Model performance was assessed with the root mean squared error (RMSE) and the corrected Akaike Information Criterion (AICc; [34]).

(3) $$RMSE=\sqrt{\frac{{\Sigma_{i=1}^{n}[{log}_{10}({N)}_{observed,i}-\log_{10}{\left(N\right)}_{predicted,i}]}^{2}}{n}}$$

(4) $$\mathrm{AICc}=nln\left(\frac{SS}{n}\right)+2K+\frac{2K(K+1)}{n-K-1}$$

In this formulation, ${log}_{10}({N)}_{observed,i}$ and $\log_{10}{\left(N\right)}_{predicted,i}$ denote the observed and model-predicted logarithmic microbial populations, respectively, while n represents the observation count. SS is the sum of squares of the residuals, and K equals the number of estimated parameters plus one. Reduced RMSE and AICc values indicate enhanced model prediction accuracy [34,35].

### 2.8. Secondary Model Fitting

The combined effects of temperature and nutritional composition on the thermal inactivation kinetics of *Salmonella* in seven milk powder varieties were examined via Response Surface Methodology (RSM; Equation (5)). The model incorporated only factors demonstrating statistical significance (*p* < 0.05). All RSM parameters were estimated using analysis of variance and response surface functions implemented in the open-source statistical software R (software R (Version 4.5.0, https://www.R-project.org/, accessed on 10 June 2025; [36]).

(5) $$D=\beta_{0}+\beta_{1}\times T+\beta_{2}\times \mathrm{Mac}+\beta_{3}\times T\times \mathrm{Mac}+\beta_{4}\times T^{2}+\beta_{5}\times {\mathrm{Mac}}^{2}$$

where Mac represents macronutrients like proteins, carbohydrates, and lipids.

### 2.9. Statistical Analysis

All experiments were conducted in triplicate. Data are presented as mean ± standard deviation (SD). Microsoft® Excel 2010 was used for primary data processing. Additionally, the model analysis described in Section 2.7 and Section 2.8 was implemented using the Python® 3.10 (Python Software Foundation, Wilmington, DE, USA). Data visualization was performed using Origin® 2017 (Origin Lab Corp., Northampton, MA, USA). Statistical significance was evaluated by analysis of variance (ANOVA) in SPSS^®^ 19 (IBM Co., Chicago, IL, USA), with a *p*-value of less than 0.05 considered significant.

## 3. Results

### 3.1. Proximate Composition Estimation

The composition of the seven powders is shown in Table 1. Different milk powders were selected to represent the macronutrient compositions in commercially available powder products. WMP (27.46% fat) and NFDM (<1% fat) were compared to evaluate the effect of fat on microbial thermal resistance WPP and CPP, with protein contents of 88.18% and 90.18% respectively, represent high-protein, low-fat and low-carbohydrate milk powders. Therefore, they can be used to assess the independent effect of protein on the thermal resistance of microorganisms Furthermore, WPP and CPP differ in protein type—the former contains whey protein while the latter consists of casein, enabling evaluation of how different protein sources affect microbial thermal resistance.

IMP, LFIM, and AAF have carbohydrate contents of 57.59%, 58.55%, and 63.24% respectively, representing high carbohydrate content milk powders while maintaining similar lipid and protein contents. Compared with other milk powders, the independent effect of carbohydrate on the thermal resistance of microorganisms can be assessed. Specifically, LFIM and AAF were strategically selected to isolate the effects of lactose and hydrolysis degree, respectively.

The differential composition of the selected milk powders enabled a comparative analysis of the effects exerted by fat, protein type, and carbohydrate source on the thermal resistance of *Salmonella*.

### 3.2. Survival of Salmonella After Reconstitution

**Figure 1:**
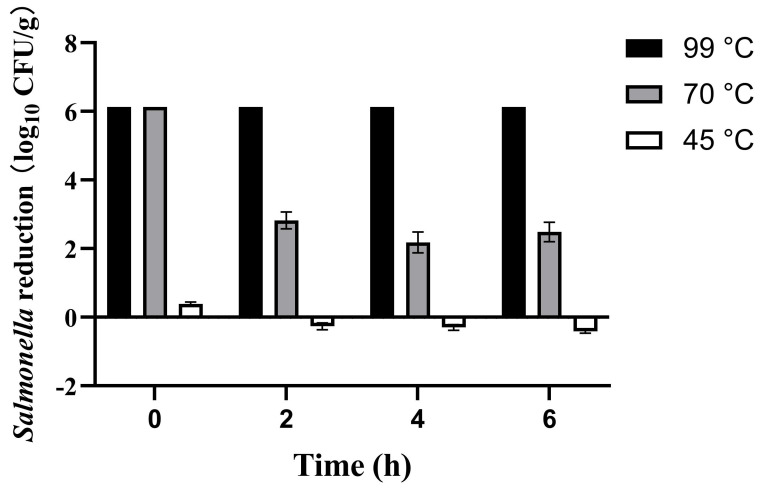
Survival of *Salmonella* in infant milk powder (IMP) after reconstituting at 45, 70, and 99 °C.

IMP inoculated with *Salmonella* was reconstituted at three distinct temperatures (45, 70, and 99 °C), corresponding to the manufacturer’s recommended preparation temperature, WHO’s advised infant formula temperature [37], and boiling water temperature, respectively. *Salmonella* reduction was investigated at 0, 2, 4, and 6 h. This is because prepared formula is not consumed immediately in practice; it typically undergoes a period of shaking for homogenization and subsequent cooling to a suitable feeding temperature. Furthermore, in outdoor scenarios such as traveling with an infant, the time between preparation and actual consumption might be prolonged. Therefore, the 0–6-h time selection help simulate potential time-temperature abuse scenarios that may occur during actual infant formula preparation and feeding practices. The results presented in Figure 1.

The experimental results demonstrated significant temperature-dependent effects on *Salmonella* reduction in IMP reconstitution. Boiling water achieved complete microbial inactivation (>6 log reduction) immediately after preparation, maintaining this effect throughout the 6-h observation. When reconstituting with 70 °C waters, the *Salmonella* on IMP was initially reduced below detection limits (>6 log). However, the *Salmonella* population was found to recover by more than 3 log at 2 h. This indicated that the *Salmonella* on IMP gradually recovered over time after this mild heat treatment. Notably, only 0.39 ± 0.06 log initial reduction was observed when reconstituting with water at a temperature of 45 °C, and then the number of *Salmonella* gradually returned to the original level over 6 h. This indicated that the manufacturer’s recommended preparation methods not only failed to inactivate *Salmonella*, but also promoted bacterial proliferation. These results suggest that *Salmonella* in IMP exhibited notable heat resistance and might resuscitate and proliferate under favorable temperature conditions.

Shi et al. [38] reported a similar finding that *Salmonella* counts in black and green tea showed no significant reduction after brewing at 25 or 55 °C for 10 min (*p* > 0.05). Significant counts reductions (*p* < 0.05) occurred at 75 °C (>4 log) and 100 °C (>8 log). However, 75 °C treatment induced viable but non-culturable (VBNC) *Salmonella*, which was generally avirulent but could resuscitate and regain virulence under favorable conditions [39]. However, high-temperature reconstitution may damage milk powder nutrients and reduce probiotic viability in infant formula [40]. They reported that probiotic viability in infant formula was reduced by 93.4% when reconstituting at 70 °C compared to 40 °C. Similar findings were observed in other foods: green beans lost 17% vitamin A after boiling at 97 °C for 60 s [41], while leeks showed 29% vitamin C loss after boiling at 94–96 °C for 90 s [42].

Contaminated IMP poses a potential risk to infants’ health under regular infant formula preparation procedures. Therefore, it is necessary to maintain its food microbial safety. However, the formula of IMP designed for different stages of infants differ in fat, protein and carbohydrate contents and types. The difference in macronutrients in different milk powder products could affect the thermal resistance of *Salmonella*, thus requiring different pasteurization conditions. Therefore, investigating the effect of micronutrition on heat resistance of *Salmonella* could build a universal model to assist in the selection of optimal treatment conditions for the pasteurization of different milk powder products.

### 3.3. Storage Stability of Powder Inoculation

**Figure 2:**
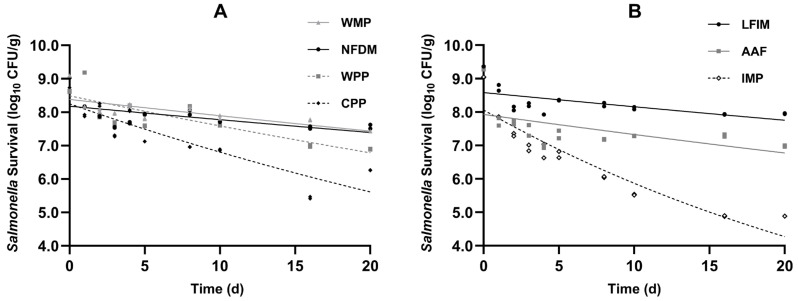
Viability of *Salmonella* population in seven types of milk powders (**A**) whole milk powder (WMP), nonfat dry milk (NFDM), whey protein powder (WPP), casein protein powder (CPP), (**B**) infant milk powder (IMP), lactose-free infant milk (LFIM), and amino acid-based formula (AAF) during storage period (25 °C). Error bars indicate 1 SD of 3 subsamples.

The survival data of *Salmonella* in four types of milk powder products and three infant formulas are presented in Figure 2. All inoculated samples showed good homogeneity and exhibited nonlinear survival curves with declining trends during 20 days.

Consistent with published reports [14,15,43,44], *Salmonella* populations in the various milk powders exhibited two distinct phases during storage, characterized by an initial rapid decline followed by a slower death rate. This pattern is commonly observed in low-water-activity foods, such as ground black pepper [45], egg white powder [46], dried basil leaves [47,48], and chia seeds [49]. This was because the desiccated environment (a_w_ < 0.70; [50]) created stress for the pathogenic bacteria, which resulted in an initial rapid decline. Through adaptation to desiccation stress, *Salmonella* developed progressively enhanced environmental resistance [51].

The initial population of *Salmonella* on inoculated WMP and NFDM was 9.09 and 9.03 log_10_ CFU/g, respectively. During 20 days of storage, the reductions of *Salmonella* in WMP and NFDM were observed to be 1.66 and 1.40 log. Similar findings were reported by Wei et al. [14]. They reported that after WMP and NFDM were adjusted to an a_w_ of 0.20, about 1.1 and 0.5 log reductions of *Salmonella* were observed, respectively. Both independent experiments demonstrated greater *Salmonella* reduction in WMP than NFDM, with no significant further decline observed during 20 days of storage. The initial bacterial counts of *Salmonella* in CPP and WPP were at a level of 8.62 and 8.68 log_10_ CFU/g, respectively, and dropped by 1.73 and 2.41 log, respectively, after 20 days of storage.

The initial population of *Salmonella* on inoculated LFIM and AAF was 9.37 and 9.31 log_10_ CFU/g, respectively. During 20 days of storage, the reductions of *Salmonella* were observed to be 1.41 and 2.32 log. The reduction in *Salmonella* in AAF was greater than that in LFIM. This phenomenon may be attributed to the release of anti-*Salmonella* active peptides in AAF, which could disrupt the bacterial membrane or virulence factors of *Salmonella*. The initial bacterial count of *Salmonella* in IMP was 9.05 log_10_ CFU/g and dropped by 4.83 log. Day et al. [52] studied *Salmonella* and Shigella survival in IMP, reporting 2.9 and 0.81 log reductions after 12-week storage, respectively. The greater reduction observed in our study might reflect differences in inoculation methods. The 20-day stability tests demonstrated that *Salmonella* remains viable in milk powders for extended periods, emphasizing the necessity of a kill step to guarantee product safety.

### 3.4. Thermal Resistance of Salmonella in Seven Types of Milk Powder

**Figure 3:**
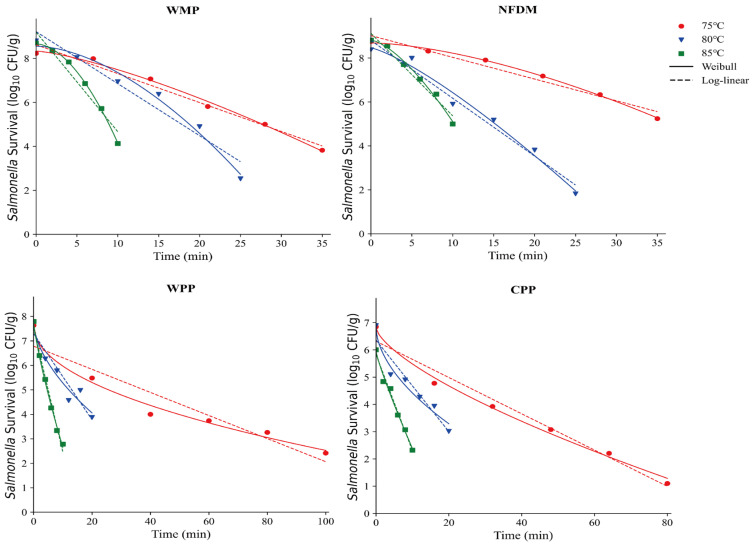
Thermal inactivation kinetics of *Salmonella* in whole milk powder (WMP), nonfat dry milk (NFDM), whey protein powder (WPP), and casein protein powder (CPP) at various temperatures.

This research investigated how macronutrient composition affects *Salmonella* thermal inactivation kinetics in seven dairy matrices: WMP, NFDM, WPP, CPP, IMP, LFIM, and AAF. The isothermal inactivation curves and thermal inactivation kinetics of *Salmonella* in WMP, NFDM, WPP and CPP are shown in Figure 3 and Table 2, respectively. The survival curve was steeper at higher treatment temperature, indicating a higher inactivation rate. For instance, the D-values of NFDM and WPP decreased significantly from 10.12 to 2.66 min and 21.21 to 1.98 min, respectively, as the treatment temperatures were increased from 75 to 85 °C.

The α-values of WMP and NFDM were greater than 1 (Table 2), indicating that their thermal inactivation curves exhibited upward convexity and the lethality rate increased over time. In contrast, the α values of WPP and CPP were less than 1, resulting in concave thermal inactivation curves, which implies a decreasing lethality rate with time. The D-values of WMP ($D_{75{}^{\circ}C}$, $D_{80{}^{\circ}C}$, $D_{85{}^{\circ}C}$) were significantly lower than those of WPP and CPP (*p* < 0.05, Table 2). Compositional analysis revealed that the protein content of WPP and CPP was higher than that of WMP (Table 1). Proteins are a key structural component of microbial cells, the proportion of protein in milk powders would influence microbial heat resistance. In this study, the higher protein contents provided additional protection to microorganisms, reducing heat-induced damage and resulting in greater heat resistance. In contrast, the lower protein content in WMP and NFDM likely made *Salmonella* more heat-sensitive, thereby affecting the shape of the thermal inactivation curves and its heat resistance. A similar finding has been observed by Jin et al. [25] that the *D*-values in high-protein matrices were larger than the *D*-values in high-fat matrices under high temperature conditions. Rachon et al. [12] demonstrated that *Salmonella* exhibits greater heat resistance than *Listeria monocytogenes* in high-protein matrices, while their heat resistance was comparable in high-salt seasoning. However, Zhang et al. [27] found that the D-values of *S. enteritidis* increased exponentially with decreasing protein contents (from 83.59 % to 31.81 %) in egg powders. This difference might be due to variations in samples, type of proteins and the different ranges of protein contents (from 10.44 % to 90.18 %) in this study.

**Figure 4:**
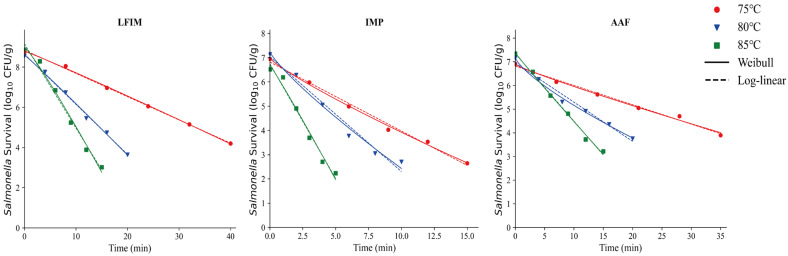
Thermal inactivation kinetics of *Salmonella* in infant milk powder (IMP), lactose-free infant milk (LFIM), and amino acid-based formula (AAF) at various temperatures.

The isothermal inactivation curves of *Salmonella* in LFIM, AAF and IMP are shown in Figure 4. These three types of infant formula exhibit notably high carbohydrate content (Table 1). Although the carbohydrate content could provide an additional energy source for microorganisms, it did not enhance their heat resistance in this study. A similar conclusion was reached by Barnes [53], the author found that under low water activity conditions, the addition of sugar did not significantly alter the survival rate of *Salmonella*. But this finding differs from some previously published studies [9,12], which suggested that carbohydrates enhance microbial heat resistance. Moats et al. [23] reported that the results with carbohydrates were variable, with mannitol, sucrose, and rhamnose providing substantial protection while glucose decreased heat resistance. These differences may be due to differences in the types of carbohydrates examined. Other research has emphasized specific types of sugars while this study focused on total carbohydrates. Moreover, the physical properties of the samples (e.g., melting behavior) further contributed to these discrepancies. Alshammari et al. [54] suggested that easily meltable samples or sugars might cover the bacteria cells, limiting migration of moisture from the headspace into the bacterial cells. In contrast, the total carbohydrates in our milk powder samples did not melt easily, likely accounting for the observed differences.

This study employed the log-linear model and the Weibull model to stimulate bacterial thermal inactivation kinetics (Figure 3 and Figure 4), with specific parameter values detailed in Table 2. Although the Weibull model provided a good fit in some cases, the log-linear model was more straightforward for linear relationships and directly yielded the *D*-values of *Salmonella* in different milk powders, which makes it the preferred fitting model for this study.

### 3.5. Influence of Temperature and Macronutrients on the Thermal Resistance of Salmonella

**Figure 5:**
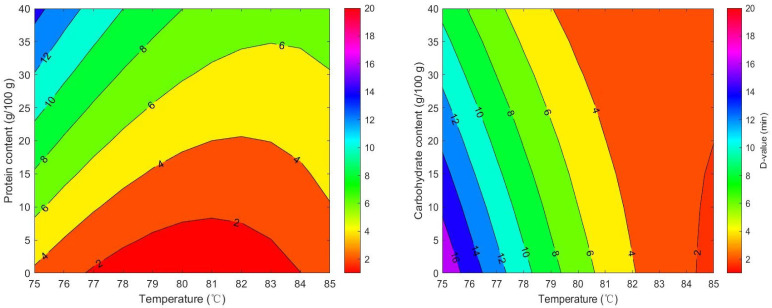
Predicted *D*-values of *Salmonella* in seven milk powder varieties based on response surface modeling of temperature and macronutrient effects.

The RSM (Figure 5) were developed to estimate *D*-values of milk powders based on their macronutrient content and temperature. The following models were derived for *D*-values:

For protein:

*D* = 708.44 − 17.669 × *T* + 1.5793 × *W_protein_* − 0.017361 × *T* × *W_protein_* + 0.10994 × *T*^2^ − 0.0010818770 × *W_protein_*^2^

RMSE = 1.28; AICc = 163.86.

For carbohydrate:

*D* = 839.87 − 19.207 × *T* − 1.9082 × *W_carbohydrate_* + 0.022597 × *T* × *W_carbohydrate_* + 0.10994 × *T*^2^ − 0.00010876 × *W_carbohydrate_*^2^

RMSE = 1.62; AICc = 185.03.

where *D* = *D*-values of seven types of milk powder, *T* = isothermal treatment temperature (°C), and *W_protein/carbohydrate_* = protein or carbohydrate contents of the sample. The effect of fat content on *D*-values was found to be non-significant. Therefore, this variable was not incorporated into the final RSM. Although some studies have demonstrated that fat exerts a protective effect on microbial thermal inactivation [27,55], no such effect was detected in milk powders. Similar findings were observed by Wei et al. [14]. Most previous studies employed pure oil phases as test media, where the continuous oil matrix can readily encapsulate bacterial cells and form a physical moisture barrier [55]. In contrast, the fat in the milk powder studied here exists in the form of dispersed droplets or embedded particles rather than a continuous oil phase, resulting in limited localized protective effects. Moreover, the synergy of complex ingredients in mike powder may weaken the protective effect of fat [12].

As the temperature increased from 60 °C to 80 °C, the *D*-value of *Salmonella* significantly decreased (*p* < 0.05), indicating that its thermal inactivation rate accelerated significantly with rising temperature. An increase in protein content (10–20%) in milk powder significantly extended the *D*-value by 1.5–2 times. This indicated that, within the test range of this study, milk powder with higher protein content showed protective effect on *Salmonella* during thermal treatment. This observation was also reported by Manas et al. [56], Rachon et al. [12], Jin et al. [25]. Various peptide mixtures, some amino acids, protein components and divalent cations contribute to the protection of the *Salmonella* cell envelope against thermal damage [28]. Milk powder proteins might bind to bacterial heat-sensitive proteins, forming complexes that stabilize critical intracellular proteins and prevent thermal denaturation [28]. Additionally, there was a statistically significant interaction between temperature and protein content. This demonstrates that the thermal inactivation process of *Salmonella* is interactively influenced by both protein content and temperature. In the low-temperature range (75–79 °C), the *D*-value curves corresponding to different protein concentrations are relatively clustered, indicating that protein content has a comparatively greater influence on *D*-values within this temperature range. One possible reason is that temperatures increase the expression of heat shock genes like rpoH. These genes might help repair damaged proteins, maintain proper protein folding and protect bacterial cells from damage [57]. In the high-temperature range (80–85 °C), the protective effect of proteins diminishes as temperature increases. The influence of protein content on D-values becomes relatively minor, while temperature becomes the dominant factor.

It has been shown that when the carbohydrate content in milk powder exceeds 50%, the *D*-value of *Salmonella* decreases by 10–15%. This indicates that higher carbohydrate content reduced the heat resistance of *Salmonella* in this study. There was no significant interaction between carbohydrate content and temperature (*p* > 0.05). This suggests carbohydrates primarily affect *Salmonella* thermal inactivation, which did not depend on temperature. The mechanisms involve osmotic pressure regulation and the formation of maillard reaction products. High carbohydrate levels increase extracellular osmotic pressure, causing cellular dehydration and disrupting metabolic activity. Additionally, the Maillard reaction is a series of complex reactions between carbohydrates and amino acids under heating conditions, and its products possess antibacterial activity, inhibiting microbial growth and reproduction [58]. Therefore, carbohydrates can accelerate the inactivation of pathogens during food thermal processing.

In summary, in the case of protein-enriched milk formulations, enhanced thermal processing parameters are required to ensure microbiological safety standards. Conversely, for carbohydrate-dominant infant nutrition products, a strategic reduction in heat treatment severity could be employed to preserve nutritional integrity while maintaining adequate *Salmonella* control measures. The developed models provide industry practitioners with critical tools for optimizing pasteurization processes, enabling data-driven selection of temperature-time parameters that ensure microbial safety while accounting for compositional variations in milk powders.

## 4. Conclusions

*Salmonella*-contaminated milk powder remains a consumption risk even when reconstituted at conventional preparation temperatures. This investigation revealed that nutritional composition exerted a significant impact on *Salmonella* thermal resistance. Across the seven milk powder formulations investigated in this study, the heat resistance of *Salmonella* was positively correlated with protein content (10.44% to 90.18%) and negatively correlated with carbohydrate content (0.35% to 63.24%). Protein conferred protection against thermal inactivation and showed a significant interaction with temperature: its effect on Salmonella D-values decreased progressively as temperature increased from 75 to 85 °C. Assessment of *Salmonella* thermal inactivation in across various milk powder formulations provides important data for optimizing pasteurization processes to ensure powdered milk safety. These universal models provide the food industry with a practical tool to predict *Salmonella D*-values under specific combinations of macronutrient composition and processing temperature.

## Figures and Tables

**Table 1:** Proximate composition (%) of whole milk powder (WMP), nonfat dry milk (NFDM), whey protein powder (WPP), casein protein powder (CPP), infant milk powder (IMP), lactose-free infant milk (LFIM), and amino acid-based formula (AAF).

Milk Powder	Ash (%)	Protein (%)	Lipid (%)	Carbohydrate (%)	Moisture (%)
WMP	6.88 ± 0.53	24.50 ± 0.15	27.46 ± 0.42	38.45 ± 1.27	2.71 ± 0.33
NFDM	9.23 ± 0.24	33.24 ± 0.22	0.82 ± 0.04	53.58 ± 0.52	3.13 ± 0.25
WPP	4.80 ± 0.17	88.18 ± 2.14	1.05 ± 0.08	0.35 ± 0.06	5.62 ± 0.26
CPP	3.45 ± 0.20	90.18 ± 1.78	0.96 ± 0.10	0.61 ± 0.07	4.80 ± 0.05
IMP	0.64 ± 0.15	10.75 ± 0.35	28.87 ± 0.66	57.59 ± 1.03	2.15 ± 0.17
LFIM	2.45 ± 0.22	10.44 ± 0.32	25.67 ± 0.58	58.55 ± 1.27	2.89 ± 0.17
AAF	1.01 ± 0.11	12.99 ± 0.29	20.15 ± 0.31	63.24 ± 1.88	2.61 ± 0.15

Values are mean ± SD of triplicates.

**Table 2:** Parameter estimates for log-linear and Weibull models for inactivation of *Salmonella* in whole milk powder (WMP), nonfat dry milk (NFDM), whey protein powder (WPP), casein protein powder (CPP), infant milk powder (IMP), lactose-free infant milk (LFIM), and amino acid-based formula (AAF).

Sample	Temperature (°C)	Weibull Model				Log-Linear Model			
		β-Value	α-Value	RMSE	AICc	*D*-Value	RMSE	AICc	Z_T_
		(Min)				(Min)			(°C)
IMP	75	2.73 ± 0.42	0.86 ± 0.07	0.091	−10.722	3.52 ± 0.16	0.135	−16.032	9.78
	80	1.54 ± 0.58	0.84 ± 0.16	0.231	0.398	2.11 ± 0.17	0.268	−7.813	
	85	1.15 ± 0.37	1.06 ± 0.21	0.242	0.978	1.06 ± 0.08	0.246	−8.845	
WMP	75	11.32 ± 1.57	1.34 ± 0.15	0.131	−6.426	7.60 ± 0.60	0.246	−8.815	8.52
	80	9.00 ± 1.80	1.73 ± 0.32	0.270	2.293	4.23 ± 0.51	0.490	−0.558	
	85	4.40 ± 0.16	1.83 ± 0.07	0.045	−19.093	2.21 ± 0.28	0.389	−3.332	
NFDM	75	16.14 ± 0.90	1.59 ± 0.10	0.056	−16.570	10.12 ± 0.97	0.227	−9.788	5.11
	80	5.71 ± 1.60	1.27 ± 0.23	0.294	3.311	3.80 ± 0.32	0.374	−3.789	
	85	4.14 ± 0.51	1.49 ± 0.19	0.120	−7.465	2.66 ± 0.25	0.243	−8.960	
WPP	75	3.46 ± 1.95	0.49 ± 0.08	0.195	−1.617	21.21 ± 3.69	0.560	1.037	3.80
	80	2.03 ± 1.54	0.58 ± 0.18	0.286	2.958	5.69 ± 0.97	0.409	−2.732	
	85	1.25 ± 0.22	0.79 ± 0.06	0.103	−9.219	1.98 ± 0.12	0.209	−10.777	
CPP	75	6.72 ± 1.81	0.69 ± 0.07	0.144	−5.270	15.02 ± 1.32	0.319	−5.713	5.40
	80	1.99 ± 1.20	0.55 ± 0.13	0.213	−0.565	5.95 ± 0.91	0.349	−4.628	
	85	2.31 ± 0.59	0.87 ± 0.14	0.145	−5.202	2.84 ± 0.19	0.161	−13.950	
LFIM	75	9.37 ± 0.72	1.05 ± 0.05	0.057	−16.297	8.65 ± 0.18	0.067	−24.350	6.50
	80	4.27 ± 0.61	1.04 ± 0.09	0.109	−8.606	4.01 ± 0.13	0.113	−18.189	
	85	2.91 ± 0.73	1.12 ± 0.16	0.229	0.297	2.39 ± 0.14	0.254	−8.446	
AAF	75	10.82 ± 1.69	0.90 ± 0.11	0.086	−11.419	12.27 ± 0.61	0.096	−20.096	7.08
	80	3.93 ± 0.70	0.75 ± 0.07	0.087	−11.353	6.05 ± 0.45	0.168	−13.414	
	85	3.31 ± 0.55	0.96 ± 0.10	0.110	−8.471	3.51 ± 0.14	0.113	−18.182	

Values are mean ± SD. RMSE = root mean square error. A single z-value of each milk powder was determined based on the 3 temperatures.

## Data Availability

The original contributions presented in this study are included in the article. Further inquiries can be directed to the corresponding authors.
